# Spread of Mink SARS-CoV-2 Variants in Humans: A Model of Sarbecovirus Interspecies Evolution

**DOI:** 10.3389/fmicb.2021.675528

**Published:** 2021-09-20

**Authors:** Christian A. Devaux, Lucile Pinault, Jérémy Delerce, Didier Raoult, Anthony Levasseur, Roger Frutos

**Affiliations:** ^1^Aix-Marseille Université, IRD, APHM, MEPHI, IHU–Méditerranée Infection, Marseille, France; ^2^CNRS, Marseille, France; ^3^Fondation IHU–Méditerranée Infection, Marseille, France; ^4^Cirad, UMR 17, Intertryp, Montpellier, France

**Keywords:** SARS-CoV-2, variant viruses, mink coronavirus, COVID-19, ACE2, NRP-1, COVID-19 vaccines

## Abstract

The rapid spread of SARS-CoV-2 variants has quickly spanned doubts and the fear about their ability escape vaccine protection. Some of these variants initially identified in caged were also found in humans. The claim that these variants exhibited lower susceptibility to antibody neutralization led to the slaughter of 17 million minks in Denmark. SARS-CoV-2 prevalence tests led to the discovery of infected farmed minks worldwide. In this study, we revisit the issue of the circulation of SARS-CoV-2 variants in minks as a model of sarbecovirus interspecies evolution by: (1) comparing human and mink angiotensin I converting enzyme 2 (ACE2) and neuropilin 1 (NRP-1) receptors; (2) comparing SARS-CoV-2 sequences from humans and minks; (3) analyzing the impact of mutations on the 3D structure of the spike protein; and (4) predicting linear epitope targets for immune response. Mink-selected SARS-CoV-2 variants carrying the Y453F/D614G mutations display an increased affinity for human ACE2 and can escape neutralization by one monoclonal antibody. However, they are unlikely to lose most of the major epitopes predicted to be targets for neutralizing antibodies. We discuss the consequences of these results for the rational use of SARS-CoV-2 vaccines.

## Introduction

Sixteen years after the identification of SARS-CoV, the causative agent of severe acute respiratory syndrome (SARS), a closely related sarbecovirus (betacoronavirus lineage 2b), named SARS-CoV-2 was isolated in Wuhan in December 2 (Guan et al., 2020; Huang et al., 2020; Zhu et al., 2020). The infection by SARS-CoV-2 is most of the time asymptomatic or moderate but in its most severe form can lead to the severe respiratory disease known as Coronavirus Disease-2019 (COVID-19) which can sometimes be lethal (Gautret et al., 2020; Koh et al., 2021). The current estimated mean fatality rate of COVID-19 is 2.07% with major disparities. In particular, the fatality rate increases with age and with the existence of underlying diseases, the most distinctive comorbidities being hypertension, coronary heart diseases, cerebrovascular diseases and diabetes (Fang et al., 2020; Zhou F. et al., 2020). In contrast to SARS-CoV (hereafter referred to as SARS-CoV-1 for the sake of clarity), SARS-CoV-2 has spread very rapidly worldwide through human-to-human transmission and was declared a pandemic in March 2020 by WHO. As per July 2021, about 188 million people have been infected worldwide and more than 4 million have died (JHU, 2021).

**FIGURE 1:**
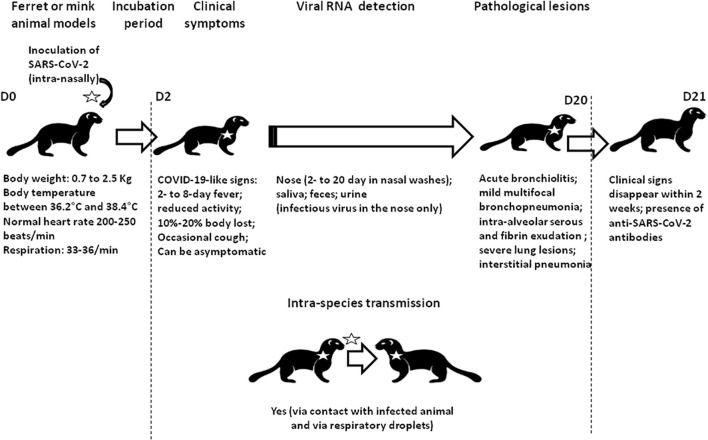
Schematic representation of the experimental infection of ferrets and minks by SARS-CoV-2. All naïve animals became febrile and most collected specimens were positive for viral RNA. The isolation of infectious viral particles was sometimes possible from nasal washes. Transmission to naïve animals was observed following direct contact with infected animals (close but in different cages). Clinical sign and histological abnormalities are summarized.

Like SARS-CoV-1, SARS-CoV-2 uses the angiotensin I converting enzyme 2 (ACE2) present on pneumocytes to enter human cells (Devaux et al., 2020; Wang and Cheng, 2020; Zhao et al., 2020). The mechanisms of interaction between the viral spike (S) protein and ACE2 were deciphered (Hoffmann et al., 2020; Lan et al., 2020; Shang et al., 2020; Wrapp et al., 2020; Yan et al., 2020). The amino acids required for this interaction were identified and the impact of their mutation ACE2 binding was assessed. This triggered intense researches on the animal origin of SARS-CoV-2. Owing to its similarities with SARS-CoV-1, initial investigations focused on bats (Forni et al., 2017; Afelt et al., 2018; Wong et al., 2019; Frutos et al., 2021a). SARS-CoV-2 shares 96.2% identity with the RaTG13, a virus sequence found in *Rhinolophus affinis* (Ra) from the Tongguan (TG) region in 2013 (Cohen, 2020; Zhou H. et al., 2020). It also shares 93.3% similarity with RmYN02 from the horseshoe bat *Rhinolophus malayanus* collected in Yunnan (Zhou P. et al., 2020), 93% similarity with RaCS203 from *Rhinolophus acuminatus* collected in Thailand (Wacharapluesadee et al., 2021), and 92.6% similarity with RshSTT182 and RshSTT200 from *Rhinolophus shameli* collected in Cambodia (Hul et al., 2021). This high sequence conservation demonstrates that SARS-CoV-2-related viruses circulate in different bats species over a large territory. With the exception of rare laboratory accidents (Normile and Vogel, 2003; Heymann et al., 2004; Watts, 2004; Della-Porta, 2008), there is no known direct transmission of coronaviruses from bats to humans. According to a virus spreading model called “spillover” (Johnson et al., 2015; Olival et al., 2017; Plowright et al., 2017), infected bats are expected to transmit their coronaviruses to an intermediate susceptible host which in turn serves as a source of virus to infect humans. A reptile (*Ophiophagus hannah*) (Callaway and Cyranoski, 2020; Zhang et al., 2020), then the Malayan pangolin (*Manis javanica*) (Liu et al., 2019; Lam T. T. et al., 2020; Zhang and Holmes, 2020) were considered as intermediaries. Pangolin coronavirus genomes display up to 92.4% sequence identity with SARS-CoV-2 (Lam T. T. et al., 2020). In particular, a high sequence similarity was reported in the receptor-binding domain (97.4% amino-acid identity). *In silico* modeling of the pangolin ACE2 receptor suggested that it could have a potential affinity for SARS-CoV-2 and a spike protein from a pangolin coronavirus was able to bind strongly to both pangolin and *Homo sapiens* ACE2 (Hsap ACE2) receptors (Wrobel et al., 2021). However, this ACE2 compatibility was also true for dozens of other species which did not attract much attention (Damas et al., 2020; Liu et al., 2020, 2021; Luan et al., 2020b). There are also arguments to exonerate the pangolin from SARS-CoV-2 transmission to humans (Andersen et al., 2020; Frutos et al., 2020a). *In silico* investigations on ACE2 polymorphism indicated that the list of SARS-CoV-2 susceptible species includes *M. javanica* (pangolin), *Macaca mulatta* (monkey), *Felis catus* (cat), *Canis lupus* (dog), *Oryctolagus cuniculus* (rabbit), *Mustela putorius furo* (ferret), *Mesocricetus auratus* (hamster), *Bos taurus* (cow), *Bubalus bubalus* (buffalo), *Capra hircus* (goat), *Ovis aries* (sheep) but not *Mus musculus* (mouse) (Blanco et al., 2020; Damas et al., 2020; Lam S. D. et al., 2020; Luan et al., 2020a, b; Qiu et al., 2020; Devaux et al., 2021). This result is a strong argument in favor of the “circulation model” in which there is no particular intermediate host but a circulation of viruses among many susceptible species including humans with no visible epizootic (Frutos et al., 2020b, 2021b). SARS-CoV-2 was found able to infect and replicate in human, non-human primate, rabbit, pig, and cat cell lines (Chu et al., 2020). *In vivo* studies indicated that monkeys are susceptible to SARS-CoV-2 and exhibit COVID-19-like symptoms (Munster et al., 2020; Rockx et al., 2020). Hamsters infected by SARS-CoV-2 also developed COVID-19-like symptoms (Chan et al., 2020; Sia et al., 2020; Song et al., 2021). SARS-CoV-2 replicated poorly in dogs, pigs, chicken, and ducks while efficiently replicating in cats (Shi et al., 2020). Both cat-to-cat (Halfmann et al., 2020; Shi et al., 2020) and human-to-cat transmission of SARS-CoV-2 (Segalés et al., 2020), was reported. Members of the *Mustelidae* family (Box 1 and Figure 1) have also been found to be susceptible to SARS-CoV-2 infection and the virus was efficiently transmitted from one infected animal to another *via* respiratory droplets (Beer, 2020; Kim et al., 2020; Richard et al., 2020; Schlottau et al., 2020; Shi et al., 2020; Shuai et al., 2021).

**Box 1. *Mustelidae* as an animal model to study SARS-CoV-2**

During the search for susceptible animals, both *M. erminea* (ermine) and *M. putorius furo* (ferret) were found to express ACE2 orthologs compatible with SARS-CoV-2 spike binding (Luan et al., 2020b; Qiu et al., 2020; Devaux et al., 2021). In addition, *M. putorius furo* is susceptible to infection with SARS-CoV-2 (Beer, 2020; Kim et al., 2020; Richard et al., 2020; Schlottau et al., 2020; Shi et al., 2020). SARS-CoV-2 is transmitted both by contact and by air between *M. putorius furo* specimen (Richard et al., 2020). Infected *M. putorius furo* usually develop mild clinical symptoms, including a fever lasting between 2 and 8 days, and an occasional cough. Viral RNA can be detected in nasal washes between 2 and 20 days after infection. Clinical signs usually disappear spontaneously within 2 weeks (Kim et al., 2020; Ryan et al., 2021). Anti-SARS-CoV-2 antibodies were detected in infected *M. putorius furo* by ELISA and neutralization assays 2–3 weeks after infection (Kim et al., 2020). Shuai et al. (2021) reported an experimental model of mink infection by SARS-CoV-2 (minks inoculated intra-nasally with 5 × 10^6^ plaque-forming units of SARS-CoV-2 HRB25 strain). SARS-CoV-2 replicated in both the upper and lower respiratory tracts and was efficiently transmitted *via* respiratory droplets. Infected minks lost between 10 and 20% of their body weight and their lungs showed severe lesions (including fibrinous necrosis of the blood vessels, intra-alveolar serous, and fibrin exudation) (Figure 1). However, none of the infected animals died. They also found that vaccinated animals can be protected against SARS-CoV-2. Whether minks display differing ACE2 and neuropilin expression levels remains to be investigated.

**FIGURE 2:**
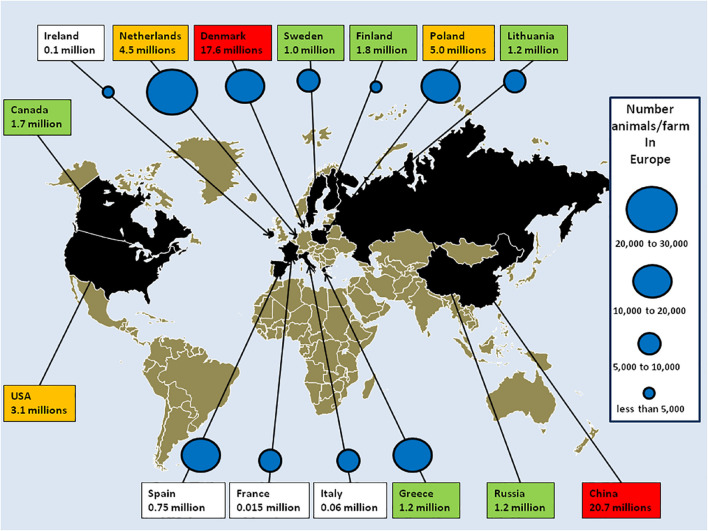
The mink fur industry. Mink stocks according to reports from the European Commission (ECDC, 2020), the fur industry (Fur Europe, 2015) and associations. China, Denmark, Poland, and Netherlands were the main producers. Red box: 10–21 million minks/year; orange: 2–10 million minks/year; green: 1–2 million minks/year; and white: below 1 million. Mass culling of minks ordered by governments after SARS-CoV-2 outbreaks in mink farms in Europe: Denmark: 17,000,000; Netherlands: 900,000; Ireland: 100,000; Spain: 92,700; Italy: 28,000; Greece: 2,500; and France: 1,000. The number of mink farms in Europe [adapted from a report from the ECDC (2020)] is indicated by blue circles (it is possible to estimate the risk of cross contamination in mink farms as a function of the density of animal populations). Fur-farming ban (year): United Kingdom 2000; Austria 2004; Croatia 2006; Serbia 2009; Slovenia and New Zealand 2013; Japan 2016; Macedonia 2017; Luxembourg, the Czech Republic and Norway 2018; and Germany and Slovakia 2019. Scheduled shutdown of mink farms: Belgium and Denmark 2023; Netherlands: 2024 (early closure 2021); France: 2025; and Bosnia and Herzegovina 2027. Outside Europe, in China in 2018 farms were mainly concentrated in the northern provinces (Shandong, Liaoning, Heilongjiang, Jilin, and Henan), and produced 20.7 million minks. In North American, 245 farms produced 3.1 million pelts in the United States and 60 farms in Canada produced 1.7 million pelt. In Russia, 22 farms produced 1.2 million minks.

During spring and summer 2020 (WHO, 2020a, b) mink farms (Box 2 and Figure 2) were found to be sites of major SARS-CoV-2 outbreaks in animals. Within a few weeks, cases of SARS-CoV-2 infection have been reported in 69 farms in Netherlands, 290 farms in Denmark, 17 farms in Greece, 13 farms in Sweden, 3 farms in Ireland, 2 farms in Lithuania, and 1 farm in Spain, Italy, Slovenia, and France (ESA, 2021). The epizootic was not limited to Europe. In mid-August 2020, the virus killed 10,000 minks across nine farms within a week in Utah (United States). In December 2020, Canada reported the death of 33 minks. The World Organization for Animal Health (OIE) set up an enhanced surveillance on *Mustelidae* deaths from SARS-CoV-2 available through the OIE WAHIS interface on a weekly basis (OIE, 2021). The first case of SARS-CoV-2 infection in a wild mink was reported in Utah (United States) (DeLiberto, 2020). This case is likely to have occurred following contact between a wild animal and an infected mink in a farm. A case of SARS-CoV-2 infection was also reported in a domesticated ferret in Slovenia (ESA, 2021). The origin of this latter case is likely to be a contact with infected humans as in the case of the mink outbreaks in northern Europe.

**Box 2. The *Mustelidae* family and mink fur farming**

The term “mink” designates *M. lutreola* from Europe and *N. vison* from America. In addition to minks, the *Mustelidae* family contains about 60 species of small carnivorous mammals (including ferrets, ermines, otters, tayras, polecats, martens, badgers, sables, and wolverines) characterized by their elongated bodies (Canuti et al., 2020). These mammals are sedentary and usually live along waterways. With the exception of the ferret, *M. putorius furo*, which has been domesticated, most *Mustelidae* species are too aggressive to be kept as pets. A century ago, wild *M. lutreola* were found widely over Europe but today only 5,000 are left in Spain, France, and the Danube delta (Karath, 2017; Skorupski, 2020). *N. vison* was introduced in Europe and Russia as fur animals in the 1920s. Animals which escaped from farms have colonized Western, Northern, and Central Europe (Lariviere, 2021). About 60 million minks are kept in farms and provide the majority of furs brought to market. The world leading producer of mink fur is China (Gong et al., 2020). In Europe, where mink farming for fur is governed by law (Council Directive 98/58/EC of July 1998), about 5,000 fur farms are located across 21 countries. At the time of the first SARS-CoV-2 outbreak on mink fur farms, the main producers in Europe were Denmark with 17,600,000 minks/year (1,533 farms), Netherlands with 4,500,000 minks/year (125 farms), Finland with 1,800,000 minks/year (914 farms), Lithuania with 1,200,000 minks/year (131 farms), and Sweden with 1,000,000 minks/year (80 farms). The report of SARS-CoV-2 outbreaks in mink farms and infection of humans with SARS-CoV-2 variants from minks shed light on an industry which had previously been very discreet about number of farms, location, density of animals in rearing, and biosafety measures (Figure 2).

**FIGURE 3:**
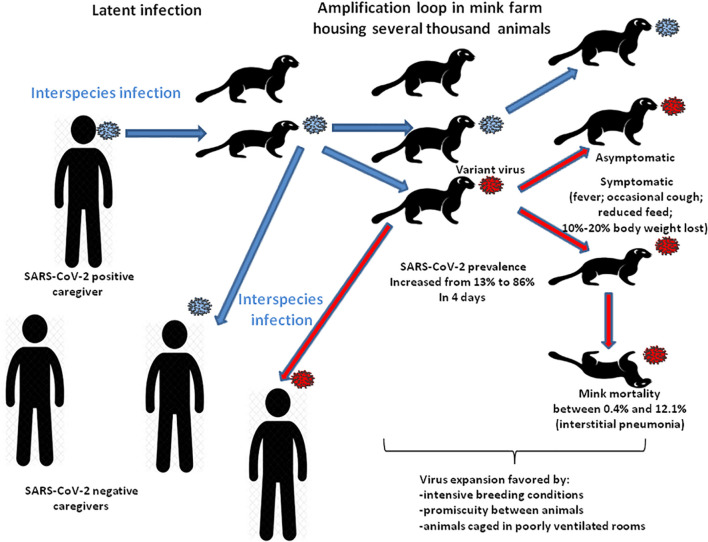
Schematic representation of the SARS-CoV-2 spread in mink farms. Infected animals reduced feed intake and lost body weight. The virus expansion in mink farms was favored by intensive breeding conditions with significant overcrowding in poorly ventilated rooms. Minks (*M. lutreola* in Netherlands and *N. vison* in Denmark) infected with SARS-CoV-2 were found to be either asymptomatic or to display signs of respiratory diseases (several animals died from interstitial pneumonia or sepsis). For fear of seeing minks-selected SARS-CoV-2 variants spreading more easily among people, of being more deadly, or having a negative impact on the deployment of anti-COVID-19 vaccines (interfering with vaccine effectiveness in humans), the Danish government decided to cull 17 million minks in more than 1,000 farms (Koopman, 2020; Larsen and Paludan, 2020; Oude Munnink et al., 2021). Countries who reported SARS-CoV-2 infected minks in farms, i.e., Netherlands, Ireland, Greece, Spain, Italy, France, United States, also started a mass slaughter.

Investigations quickly demonstrated that minks could transmit variants to humans (Box 3 and Figure 3). A mink-selected SARS-CoV-2 variant with four mutations H69del/V70del, Y453F, I692V, and M1229I, was found in humans in the northern region of Jutland. Preliminary results suggested that this variant displayed weak reactions to human neutralizing anti-SARS-CoV-2 antibodies. However, the limitation of the neutralization assay is that it is highly dependent on the 50% tissue culture infectious dose (TCID_50_), concentration of antibodies in the plasma tested, plasma dilution, and epitope-specificity of the antibodies which can vary from one individual to another. To date there is little evidence that these mutations are of particular concern. However, within just a few months, the status of *Mustelidae* changed from that of an animal model to a source of variant viruses threatening humans.

**Box 3. Epidemiology of SARS-CoV-2 in captive minks**

In mid-April 2020, an increased mink mortality was observed in two mink farms located in the province of North Brabant (Netherlands), housing 13,700 and 7,500 animals (*M. lutreola*), respectively. The median mink mortality was 0.45% (Boklund et al., 2021). Necropsied minks mainly showed interstitial pneumonia. The investigation of SARS-CoV-2 outbreaks in Netherlands indicated the introduction of the virus into farms by humans and subsequent transmission among minks (Oreshkova et al., 2020; Boklund et al., 2021; Figure 3). A very rapid spread of the virus was also reported in farms in Denmark, with an increase in virus prevalence from 13 to 86% within 4 days in one farm, suggesting that a greater fitness of the variant for minks increased the transmission rate (Hammer et al., 2021). SARS-CoV-2 infection was then reported in hundreds of farms in Europe, United States, and Canada. Some 170 mutations were identified (Mallapaty, 2020). SARS-CoV-2 mink variants, e.g., cluster 5 variant, were found in staff members, showing evidence of bidirectional infections with up to 68% infection in farm employees (ECDC, 2020; Molenaar et al., 2020; Oreshkova et al., 2020; Oude Munnink et al., 2020; Hammer et al., 2021). International health authorities reported that a mink-selected SARS-CoV-2 variant infecting people in Jutland displayed weak reactions to human neutralizing anti-SARS-CoV-2 antibodies. There is currently little evidence that these mutations are of particular concern (Frutos and Devaux, 2020). However, the decision was taken to cull all of the 17 million minks in Denmark. This decision ignored that the real drivers of epidemics and pandemics are human activities. In the Danish case, it was the presence of SARS-CoV-2-positive employees who were in contact with animals caged at a very high density, a condition that facilitated the spread of the virus among animals and then among SARS-CoV-2 seronegative employees (Frutos and Devaux, 2020; Mallapaty, 2020; Boklund et al., 2021).

In this study, we considered minks as a model for studying the human/animal interface and tried to look back at the SARS-CoV-2 infection of minks in farms to question the selective sweeps of the virus in infected *Mustelidae*, to analyze which SARS-CoV-2 variant viruses passed from *Mustelidae* to humans, and to assess the risk of mink-selected SARS-CoV-2 variants to humans and to vaccine programs.

## The Angiotensin I Converting Enzyme 2 Receptor in Minks

**FIGURE 4:**
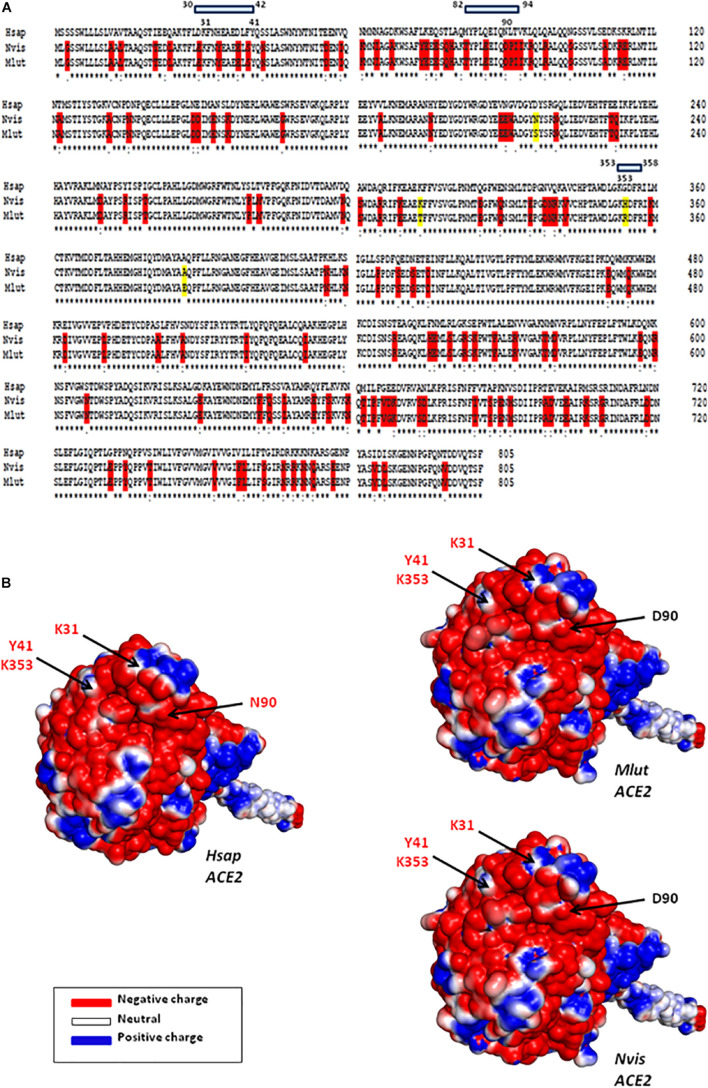
Mink ACE2 sequences alignment. **(A)** Clustal Omega multiple sequence alignment (EMBL-EBI bioinformatic tool; Copyright EMBL 2020) was used to compare the ACE2 protein sequences of *H. sapiens* (Hsap) and minks *N. vison* (Nvis) and *M. lutreola* (Mlut). The amino acids that differ between human and minks are shown in red and those which differ between minks are shown in yellow. Some of the amino acids important for viral tropism are indicated in blue (previous studies showed that amino acid residues 31, 41, 90, and 353 are important for viral spike binding). **(B)** 3D model of ACE2 designed using the Phyre2 server (this model lacks the cytoplasmic tail of ACE2). An electrostatic potential surface (red: negative charge; blue: positive charge) was generated using PyMOL 1.8.0 and APBS tool plugin. The location of amino acids 31, 41, 90, and 353 is indicated by arrows.

We and others previously investigated the ability of ACE2 from different species to interact with SARS-CoV-2 by comparing the sequence of ACE2 orthologs with that of the Hsap ACE2. These *in silico* studies predicted that ACE2 orthologs from both *Mustela erminae* (Qiu et al., 2020) and *M. putorius furo* (Luan et al., 2020b; Devaux et al., 2021), could serve as a receptor for SARS-CoV-2. At that time, only the ACE2 sequence of *M. erminae* (Merm ACE2) and *M. putorius furo* (Mput ACE2) were available in the databases. The Mput ACE2 sequence expresses the K31, Y41, and K353 amino acids required for interaction with the SARS-CoV-2 spike. A D90 is present instead of a N90 residue which is considered to be important for interactions (this position is glycosylated in the Hsap ACE2). More recently, the ACE2 sequences of both *Neovison vison* (Nvis ACE2) and *Mustela lutreola* (Mlut ACE2) were made available through the NCBI database. A sequence alignment of ACE2 was performed using Clustal Omega 1.2.4^1^ and ACE2 sequences: *H. sapiens* Hsap ACE2 (Q9BYF1), *N. vison* Nvis ACE2 (QPL12211), *M. lutreola* Mlut ACE2 (QNC68911), *M. erminea* Merm ACE2 (XP_032187679), *M. putorius furo* Mput ACE2 (XP_004758943), and *Mustela nigripes* Mnig ACE2 (QNC68914). As shown in Figure 4, the ACE2 sequences from Nvis and Mlut shared only 83.73 and 83.48% amino acid identity with the Hsap ACE2, respectively, while the ACE2 sequences from Nvis and Mlut displayed 99.51% similarity one to the other (Figure 4A). The similarity between Hsap ACE2 and mink ACE2 dropped to 63.34% in the region described to be involved in the interaction with the SARS-CoV-2 spike protein (regions 30–41, 82–93, and 353–358). Despite the difference in amino acids 131 and 133 between Nvis ACE2 and Mlut ACE2, respectively, and the Hsap ACE2, the K31, Y41, and K353 amino acids required for interaction with the SARS-CoV-2 spike protein are conserved. The ACE2 receptor from minks displays a D90 instead of a N90 residue, a change already observed with the Mput ACE2. It is therefore likely that the affinity of the SARS-CoV-2 spike protein for Nvis ACE2 and Mlut ACE2 differs from that for Hsap ACE2. This is interesting because previous studies on SARS-CoV-2 variants have only focused on their affinity for Hsap ACE2.

**FIGURE 5:**
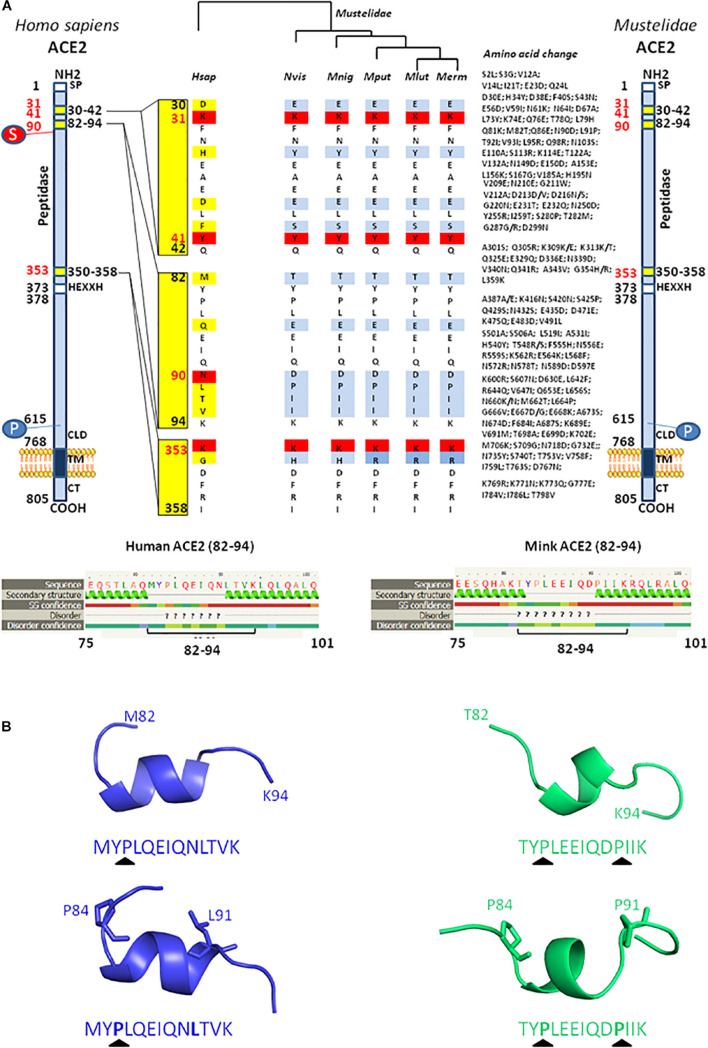
Comparison between *H. sapiens* and different *Mustelidae* ACE2 proteins. **(A)** A schematic representation of the cell surface of the Hsap ACE2 molecule and its major domains is shown on the left side of the figure while the representation of the *Mustelidae* ACE2 is displayed on the right side of the figure. Amino acid changes compared to the human sequence are listed. The amino acid position is shown in black. The most important amino acids for SARS-CoV-2 interaction are in red. S, sugar; P, phosphorylation. The comparison of the *H. sapiens* (Hsap) ACE2 with five different ACE2 sequences from *Mustelidae*, i.e., *N. vison* (Nvis), *M. nigripes* (Mnig), *M. putorius furo* (Mput), *M. lutreola* (Mlut), *M. erminea* (Merm) was performed using Clustal Omega. The figure illustrates the homology (white or red) and difference (light blue or deep blue when there is a difference in amino acid usage between *Mustelidae*) in the three regions (30–42, 82–94, and 353–358) involved in SARS-CoV-2 binding (middle panel). **(B)** Local prediction of the 82–94 amino acids peptide secondary structure using the Phyre2 server and three-dimensional folding using PeP-FOLD algorithm.

Due to the high number of amino acid differences between Hsap ACE2 and both Nvis ACE2 (131 amino acid changes/805) and Mlut ACE2 (133 amino acid changes/805), we questioned whether these differences could lead to electrostatic potential surface modifications. The ACE2 protein structure modeling was performed using the Phyre2 server (Kelley et al., 2015). The PyMOL 1.8.0 software^2^ and the Adaptive Poisson–Boltzmann Solver (APBS) tools plugin^3^ were used to generate electrostatic potential surfaces of the human ACE2, Mlut ACE2, and Nvis ACE2 orthologs. The red color indicates an excess of negative charges while white and blue indicate neutral and positively charged surfaces, respectively. The prediction of the peptide structure was performed using the PEP-FOLD Peptide Prediction Server-RPBS^4^ as previously described (Thévenet et al., 2012; Shen et al., 2014). As shown in Figure 4B, although some spots appear with different charges, the extent of electrostatic potential surface modifications remains moderate. However, near the K31 amino acid, which is a key residue for SARS-CoV-2 binding, a spot of positively charged surfaces is found in the Nvis ACE2 and Mlut ACE2 while absent in the Hsap ACE2. To further explore the ACE2 polymorphism between human and minks, we compared the ACE2 sequences from five *Mustelidae* to that of Hsap ACE2. As shown in Figure 5A, the ACE2 sequences from *Mustelidae* were highly conserved in the three regions (30–42, 82–94, and 353–358) previously determined to interact with SARS-CoV-2. We only found that the H354 residue for Nvis, Mnig, and Mput ACE2 was replaced by R354 in Mlut ACE2 and Merm ACE2. The most important changes were found in the 82–94 region with the N90D mutation changing the capacity for sugar modification and the L91P mutation in which a proline in the *Mustelidae* ACE2 sequence is likely to modify the 3D structure. The secondary structure prediction also suggested that the 82–94 region is a disordered structure surrounded by two alpha helices. The 3D modeling also suggested that human and mink ACE2 (82–94) may fold differently (Figure 5B). Whether the SARS-CoV-2 spike displays a higher or lower affinity for the *Mustelidae* ACE2 when compared to Hsap ACE2 remains to be investigated. Moreover, mutations in the SARS-CoV-2 spike protein might have been selected in minks to increase the fitness of the virus to bind the mink ACE2.

## The Neuropilin 1 Receptor in Minks

Beside ACE2, another cell surface molecule, neuropilin 1 (NRP-1), is known to bind to furin-cleaved substrates, potentiating thus the SARS-CoV-2 infectivity (Cantuti-Castelvetri et al., 2020; Daly et al., 2020). NRP-1 is expected to increase viral entry into ACE2-positive/NRP-1-positive cells of the olfactory epithelium through interaction with a C-end rule (CendR; R/K/XXR/K) terminal motif RRAR_*OH*_ in the SARS-CoV-2 spike protein which becomes exposed after cleavage of the polybasic furin-type peptidase site at the S1–S2 junction. NRP-1 is a transmembrane glycoprotein of 923 amino acids, and the interaction with CendR is mediated by the two Factor V/VIII homology (β1/β2) domains which consist of two tandem regions of approximately 150 amino acids each (Pellet-Many et al., 2008; Teesalu et al., 2009; Haspel et al., 2011).

We analyzed *in silico* the neuropilin orthologs in different animal species (Supplementary Figure 1). The sequences alignment of NRP-1 was performed with Clustal Omega 1.2.4 using the following sequence: *H. sapiens* Hsap NRP-1 (O14786), *N. vison* Nvis NRP-1 (CCP78142), *M. erminea* Merm NRP-1 (XP_032205217), *M. putorius furo* Mput NRP-1 (XP_004774343), *Lontra canadensis* Lcan NRP-1 or river otter (XP_032693696), and *Enhydra lutris kenyon* Elut NRP-1 or sea otter (XP_022371398). The NRP-1 protein was fairly conserved among species. The Mput NRP-1 showed 96.75% identity with the Hsap NRP-1 (30 amino acid changes/923). The amino acids S346, E348, T349, and K373 in β1, important for CendR binding, were conserved. A K359R mutation was observed at a position considered important for CendR binding but the charge (positive) remained unchanged. R513, K514, and K516 in β2, important for CendR binding, were also conserved. We next compared the NRP-1 from different *Mustelidae* including *M. putorius furo*, *M. erminea*, *N. vison*, *L. canadensis*, and *E. lutris kenyon* (Supplementary Figure 2). The NRP-1 protein displayed 99.13% identity (8 amino acid changes/923). These data suggest that all NRP-1 receptors are likely to bind to the CendR RRAR_*OH*_ motif of SARS-CoV-2 with similar affinity.

## Mutations in the Mink SARS-CoV-2 Genome

**FIGURE 6:**
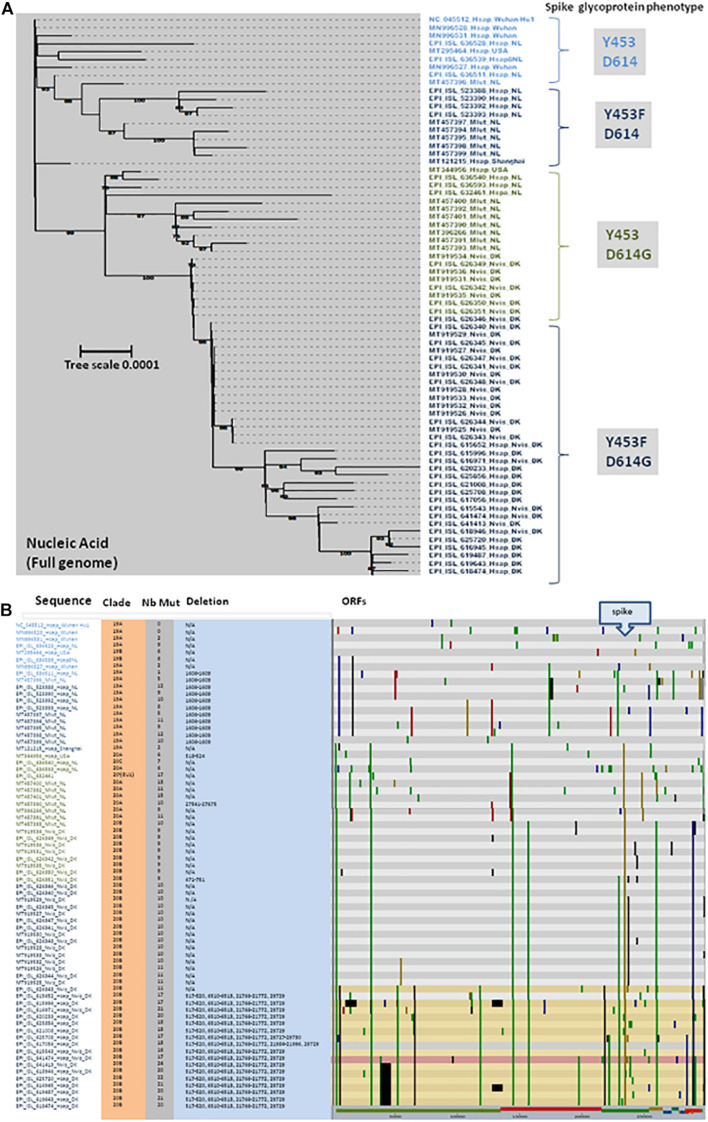
**(A)** Maximum likelihood phylogenetic tree of different genomic SARS-CoV-2 sequences. Sample names are built with the GISAID or GenBank accession number followed by a four-letter code (Mlut, Nvis, and Hsap) identifying the species followed by a code indicating the geographical origin of the sample (Wuhan: Wuhan, China; NL: Netherlands; DK: Denmark; and USA: United States). This analysis compares the full genome sequence from minks (Mlut: *M. lutreola* or Nvis: *N. vison*) and human (Hsap). The main spike phenotype (amino acid substitution) of each group of SARS-CoV-2 is indicated on right side of the figure. Four different groups where identified: Y453/D614, Y453F/D614, Y453/D614G, and Y453F/D614G. **(B)** Location of genomic mutations and deletions on the complete nucleotide sequence from different isolates of SARS-CoV-2. Color code: red, mutation to A; blue, mutation to C; green, mutation to T; yellow, mutation to G; light gray, deletion; and dark gray, not covered. Nb Mut, number of mutations; ORFs, open reading frames.

As a result of international efforts around whole genome sequencing (WGS) of SARS-CoV-2 strains and the rapid availability of sequences on the GISAID and GenBank databases, it was possible to analyze the phylogeny and evolution of these viruses in humans and other species almost in real time (GISAID, 2021). We selected more than 40 genomic sequences of SARS-CoV-2 infecting minks and almost 30 sequences from SARS-CoV-2 infecting humans to performed a phylogenetic analysis. Full genomic sequences were aligned with MAFFT v7.310 and a phylogenetic tree was built using iqtree2.1.2 under the GTR+R model and 1,000 ultrafast bootstraps. The phylogenetic tree was visualized with iTOL (Letunic and Bork, 2016). Nextclade was used to identify the position of the mutation in the genome compared to the SARS-CoV-2 original Wuhan Hu1 strain (NC_045512). Accession numbers are indicated in the figure (e.g., MN996528 or EPI_ISL_636528). In order to specify the origin of the sample, a simple code was provisionally added after the accession number to specify whether it is a human sample (Hsap) or a sample from *M. lutreola* (Mlut) or *N. vison* (Nvis), followed by a location code (e.g., NL for Netherlands; DK for Denmark). The full-length mink SARS-CoV-2 sequences were selected following a typing based on the spike protein mutations. Sequences were grouped according to these spike protein mutations and for each group several sequences were selected to represent different countries and hosts. When only one or two sequences were present in a given group, all were considered. These sequences are distributed into four distinct groups (Figure 6A). These analyses first showed that there was no segregation between the human SARS-CoV-2 and mink SARS-CoV-2, but each time a mix between viruses from minks and humans. This indicated that the different types of variants can infect both species indistinctly. Although mutations were spread across different open reading frames of the SARS-CoV-2 genome, the frequency of mutations was higher in the gene encoding the spike glycoprotein (Figure 6B). This suggests that when the virus moves from one species to another (e.g., from humans to minks), the selective pressure (mainly the immune response against the most exposed viral proteins and particularly the spike) on the viral population in the “new environment” (new host), tends to lead to an increased frequency of mutations. It is likely that when a mutation increases the viral fitness, it might be conserved after a transfer back to other species (e.g., from minks to humans). This simple evolutionary process contributes to the generation of a large panel of variants. Moreover, SARS-CoV-2 adapted to minks can be found in humans and are relatively different from the strains which hitherto circulated in this species. This observation supports the “circulation model” as the main driving force behind the expansion of the range of virus hosts.

## Mutations in Mink SARS-CoV-2 Spike (Genome and Protein)

**FIGURE 7:**
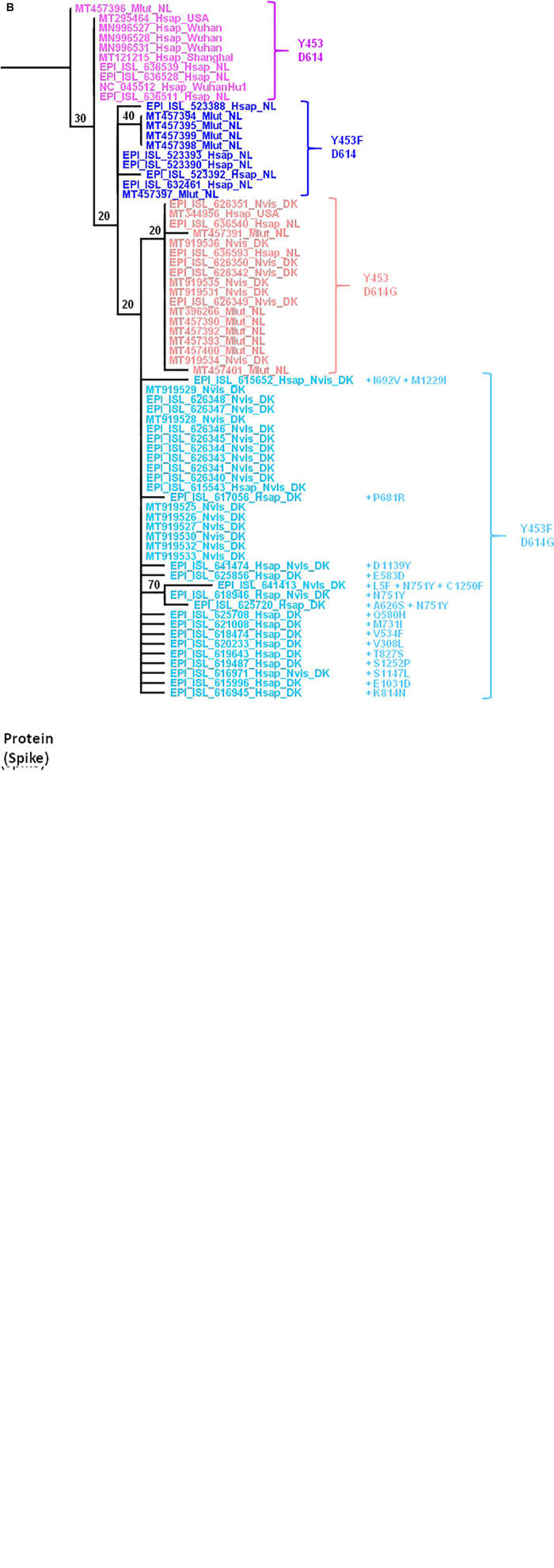
**(A)** Maximum likelihood (GTR+R) phylogenetic tree of the SARS-CoV-2 spike gene from different isolates. Sample names are built with the GISAID or GenBank accession number followed by a four-letter code (Mlut, Nvis, and Hsap) identifying the species followed by a code indicating the geographical origin of the sample. The tree was rooted using the spike gene and protein sequence of the Wuhan Hu1 SARS-CoV-2 strain. **(B)** Maximum likelihood (LG) phylogenetic tree of SARS-CoV-2 spike glycoprotein from the different isolates.

We focused on the mutations observed within the viral spike both at the genomic (Figure 7A) and protein (Figure 7B) level since it is the main target for the human vaccine strategies. Open access tools make it possible to quickly visualize the evolution of strains such as the SARS-CoV-2 cluster 5 in minks carrying the Y453F mutation in the spike protein (Nextstrain, 2021). The Y453Fmutation, found both in Denmark and Netherlands, was selected in minks after infection. It may thus confer a selective advantage in mink-to-mink transmission through a better affinity for the mink ACE2 and/or decreasing sensitivity to the neutralizing immune response. According to the European CDC, it has also been detected sporadically in SARS-CoV-2 sequences originating from Russian, South African, Swiss, and United States patients with no apparent link to the variants found in Denmark or Netherlands (ECDC, 2020). This circulation of mink-adapted viruses in countries where no mink farms are present is the result of human-to-human transmission. The D614G mutation was previously reported as a variant having emerged in humans and conferring higher affinity for the ACE2 receptor (Korber et al., 2020). The alignment of the full spike genes available through the GISAID and GenBank databases was performed with MUSCLE (Edgar, 2004) in the SeaView package (Gouy et al., 2010). The genomic tree was built using the maximum likelihood method under the GTR+R model with 500 bootstrap repeats. The protein tree was built using the maximum likelihood method with the LG model with 500 bootstrap repeats. The mink viruses can be separated in four different groups according to the amino acid expressed at positions 453 and 614. In Group 1 (e.g., MT457396_Mlut_NL), mink SARS-CoV-2 from Mlut in Netherlands displayed both Y453 and D614. This mink virus shared homology with human SARS-CoV-2 isolated in Asia, Netherlands, and United States. It could be considered a human-originating D clade virus. In Group 2 (e.g., MT457394_Mlut_NL), the SARS-CoV-2 sequences from Mlut in Netherlands and Nvis in Denmark shared homology with human SARS-CoV-2 strains from Netherlands and United States. These viruses, which expressed a wild type D614 and a mutated Y453F amino acid, could be considered as D clade viruses of human origin having been transferred to minks where they acquired the Y453F mutation and then re-infected humans with a conserved Y453F mutation. In Group 3 (e.g., EPI_ISL_626351_Nvis_DK), the SARS-CoV-2 sequences from Mlut in Netherlands shared homology with human SARS-CoV-2 from Netherlands only. They could be considered G clade viruses from human origin. These viruses conserved the human consensus Y453 under the mink immune system selection, suggesting a recent introduction. These viruses were apparently not circulating in humans in Denmark. Sequences from the Group 4 (e.g., EPI_ISL_626348_Nvis_DK), SARS-CoV-2 from Nvis in Denmark shared homology with human SARS-CoV-2 from Denmark only. They could be considered G clade viruses from human origin likely to have acquired the Y453F mutation under mink selection before being reintroduced into humans. This group, which only included virus sequences from Nvis with no equivalent found in Mlut minks, showed the highest frequency of mutations in the spike protein. The consequences in terms of ACE2 affinity, viral replication and cytopathic effect should be further explored. None of these mutations mapped onto the receptor binding domain. Most fell into highly structured regions and could possibly influence the spike trimer formation.

## Three-Dimensional Location of Mutations in the Spike Protein: Relationship With Angiotensin I Converting Enzyme 2 Binding and Linear Epitopes

**FIGURE 8:**
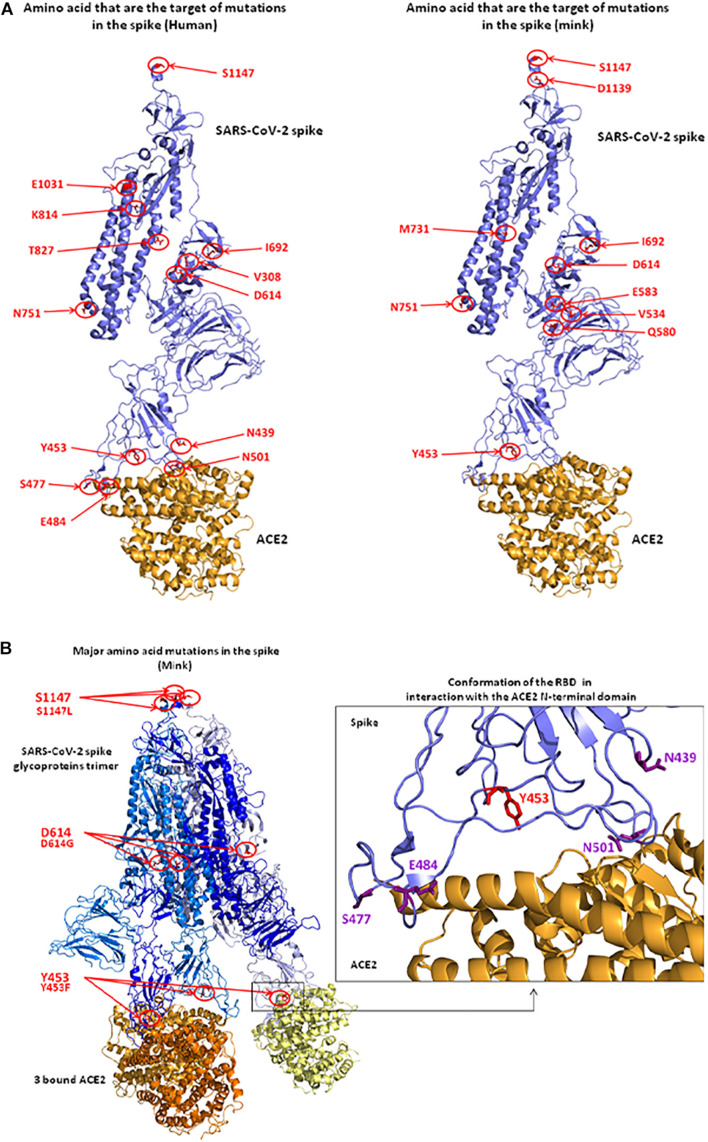
3D structure of the SARS-CoV-2 spike protein complexed with ACE2. **(A)** Positions of observed mutant residues in human (left) and mink (right) SARS-CoV-2 spike proteins. In the published 3D structure (PDB: 7A98) (Benton et al., 2020), the spike glycoprotein is colored in blue and the bound ACE2 receptor is colored in orange. The main mutant amino acids are colored in red and their positions are indicated with red circles and arrows. **(B)** Representation of the SARS-CoV-2 spike trimer (SARS-CoV-2 sequence from mink) in interaction with ACE2 molecules (left). Representation of the conformation of RBD in interaction with the N-terminal domain of ACE2. The Y453 amino acid that was changed to F453 after the infection of minks (right).

**FIGURE 9:**
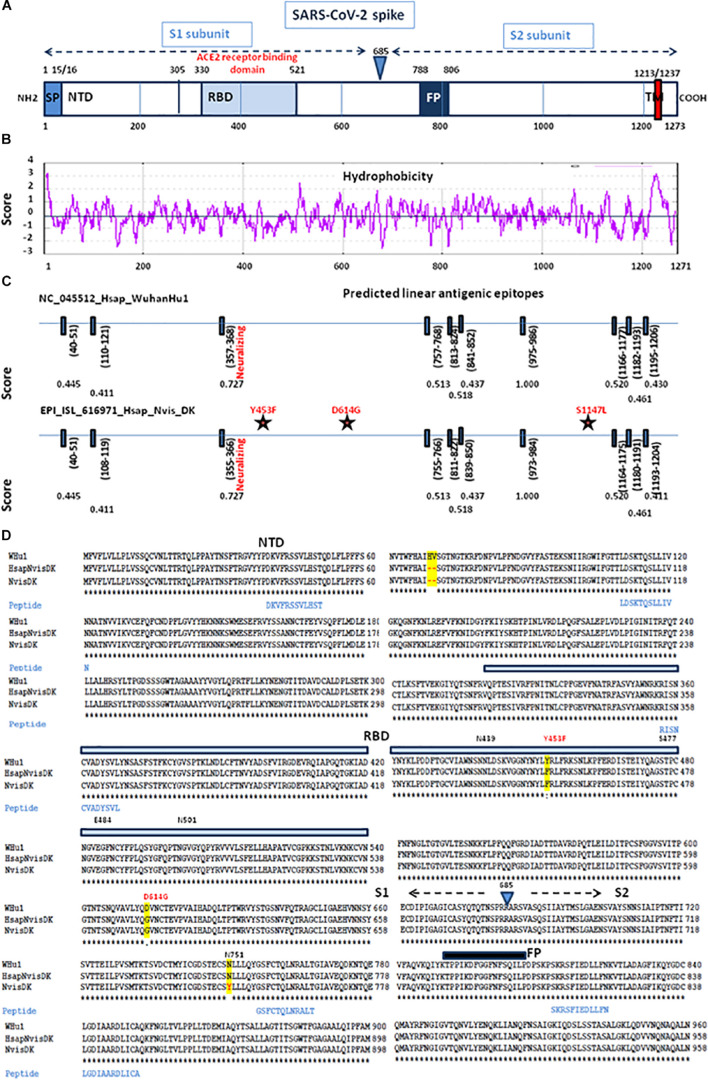
Comparative antigenicity of human and mink SARS-CoV-2. **(A)** Schematic representation of the SARS-CoV-2 spike protein domains. SP, signal peptide; NTD, N-terminal domain; RBD, receptor (ACE2) binding domain; TM, transmembrane domain. **(B)** Prediction of protein hydrophobicity according to Kite and Doolittle using the ProtScale program. The score of hydrophobicity is indicated. **(C)** SVMTrip algorithm prediction of linear antigenic epitopes (12 amino acids scan) in the spike protein of SARS-CoV-2 (NC_045512_Hsap_WuhanHu1 isolate versus EPI_ISL_616971_Hsap_Nvis_DK). The location of the predicted linear epitopes is indicated as well as the score of antigenicity (1.000 being the highest score). The stars indicate the mutations found in the mink SARS-CoV-2 variant EPI_ISL_616971_Hsap_Nvis_DK. **(D)** Multiple sequence alignment of the first 960 amino acids of the spike protein sequence (S1 and the proximal S2) from the NC_045512_Hsap_WuhanHu1 isolate versus EPI_ISL_616971_Hsap_Nvis_DK and EPI_ISL_641413_Nvis_DK samples and location of the predicted linear B-cell epitopes. The amino acids differing between the three sequences are highlighted in yellow. The location of the variants Y453F and D164G is shown in red. NTD, N terminal domain; RBD, receptor binding domain; FP, fusion peptide.

Based on the 3D modeling of the SARS-CoV-2 spike-human ACE2 interactions we visualized the location of amino acids described as major mutation sites in the SARS-CoV-2 variants infecting humans, i.e., the S477N, E484K, and N501Y/T mutations known to characterize the Marseille-4 variant (France), the 20I/501Y.V1 variant (United Kingdom), and the 20H/501Y.V2 variant (South Africa) (Figure 8A, left panel). The 3D structure of the ACE2-bound spike protein from SARS-CoV-2 was retrieved (PDB: 7A98) (Benton et al., 2020). PyMOL 1.8.0 (see text footnote 2) was used to highlight mutant amino acids in both human and mink S proteins and to generate 3D pictures. The three main amino acid positions, i.e., Y453, D614, and S1147, specific to the spikes of SARS-CoV-2 infecting minks were also visualized (Figure 8A right panel). Y453F was the only mutation located in an RBD region which could directly change the affinity of the S protein-ACE2 interaction by reducing the clash of polar groups (Figure 8B). The other two, i.e., D614 and S1147, were far from the site of interaction. The D614G mutation which became dominant in human SARS-CoV-2 isolates, was reported to increase viral infectivity (Plante et al., 2021). This D614G mutation would facilitate the formation of trimeric S protein complexes permitting thus a stable conformation which in turn led to a higher affinity for the ACE2 receptor (Korber et al., 2020). In addition, mutations or deletions such as H69del/V70del in the N-terminal domain (NTD) of the spike could change the early interaction of SARS-CoV-2 with the gangliosides lipid rafts of the plasma membrane independently from the RBD binding to ACE2 (Fantini et al., 2021). We analyzed the hydrophobicity of the NC_045512_Hsap_WuhanHu1 S protein (sequence length 1,271 amino acids) and the S proteins of viruses infecting minks (EPI_ISL_641413_Nvis_DK and EPI_ISL_641474_Hsap_Nvis_DK) to determine which regions of the spike were hidden or exposed to antibodies (Figures 9A,B). The hydrophobicity of the NC_045512_Hsap_WuhanHu1 S protein was determined according to the Kyte and Doolittle model using ProtScale (Expasy^5^). The linear antigenic epitopes (12 amino acid cut off; score from 0.411 to 1.000) for SARS-CoV-2 strains NC_045512_Hsap_WuhanHu1, EPI_ISL_616971_Hsap_Nvis_DK, EPI_ISL_641413_Nvis_DK, and EPI_ISL_641474_Hsap_Nvis_DK, were predicted using the SVMTriP program available through the University of Nebraska-Lincoln.^6^ Sequence alignments of both the SARS-CoV-2 spike and ACE2 proteins were performed using Clustal Omega 1.2.4. The three SARS-CoV-2 spike sequences were predicted to display the same 10 linear epitopes (Figure 9C). None of these putative linear epitopes overlapped with any of the three Y453, D614, and S1147 positions for which mutations were reported in mink SARS-CoV-2 variants (Figures 9C,D). Out of the 10 predicted antigenic peptides, only one was found to map within the RBD. It might be a target for neutralizing antibodies. According to this observation, it can be hypothesized that when SARS-CoV-2 variants from minks re-infect humans, they should remain sensitive to neutralization.

## Discussion

SARS-CoV-2 was reported in Wuhan (Guan et al., 2020; Huang et al., 2020; Zhu et al., 2020) in December 2019, but it was probably already circulating in China several weeks before, likely in October and November 2019 (Andersen et al., 2020; Frutos et al., 2020a). WGS is the best tool for understanding the transmission dynamics of SARS-CoV-2 worldwide. However, this approach requires access to sequence platforms providing real-time sequencing and high-speed data analyses. Costs remain high and this strategy is mostly reserved for analyzing viruses infecting humans (Levasseur et al., 2020; Oude Munnink et al., 2020). However, since SARS-CoV-2 can easily pass from one species to another (e.g., from humans to minks and then back to humans), a similar surveillance of viruses in species susceptible to SARS-CoV-2 and in contact with humans is equally important. ACE2 and TMPRSS2 are key factors in SARS-CoV-2 infection process, but mutations in ACE2 seem to have a more disruptive effect in ACE2 than in TMPRSS2 (Lam S. D. et al., 2020). When introduced into a new species, the virus is likely to acquire mutations that ensure efficient viral spread by optimizing interactions with host cell factors like ACE2. Many of the SARS-CoV-2 genomes characterized in minks were identical to that of humans. However, mutations also occurred in mink-SARS-CoV-2 and may be the result of adaptation during viral transmission between minks. Although some mink-derived variants can infect humans they appeared to be less lethal and infective compared to those in humans (Konishi, 2020). Very recently, it was reported that viral entry into certain SARS-CoV-2 susceptible cell lines was reduced when the mink-derived Y453F mutation was combined with H69Δ, V70Δ, I692V, and M1229I (Hoffmann et al., 2021). It can also be hypothesized that the mink-derived variants that have mutated further among minks and are more lethal for minks, kill the host more quickly and will be transmitted to humans less often.

Fear of human infection with SARS-CoV-2 variants selected in minks has encouraged laboratories to perform WGS on mink SARS-CoV-2 strains, demonstrating that the transfer is bidirectional between humans and minks, each being a source of infection (Oude Munnink et al., 2020). It is necessary to monitor what occurs in infected animals in addition to what happens in infected humans in order to adapt intervention strategies and design protective vaccines (Poland et al., 2020). Although it cannot be completely ruled out that the outbreak of SARS-CoV-2 in certain mink farms could be associated with the introduction into the farm of a wild animal carrier, e.g., wild *Mustelidae* which sometimes enter farms to steal food (Trebbien et al., 2014), it is much more likely that it is linked to transmission by staff members responsible for cage maintenance. Indeed, the first sequences of mink SARS-CoV-2 were derived from a human SARS-CoV-2. SARS-CoV-2 mutations selected in minks appeared only afterwards. A total of 68% of mink farm employees and/or contacts were positive for SARS-CoV-2 infection (Oude Munnink et al., 2020). However, commercial mink farming around the world has become an economical and animal welfare dilemma. Until 2020, the fur industry employed over one million people worldwide and generated a profit estimated at €35 billion. In Europe, about 100,000 people worked in the fur sector and an 42,669,000 mink pelts were sold annually representing an income of €994 million (Fur Europe, 2015). For instance, the fur sector in Netherlands employs around 1,200 full-time and 400 part-time persons. Due to the increase in consumer demand for animal welfare and to activist protests against an industry presented as cruel (more than 50 minks must be killed to make a single fur coat), a fur farming ban was requested. Many countries decided to shut-down this industry, i.e., United Kingdom, Austria, Croatia, Serbia, Slovenia, New Zealand, Japan, Macedonia, Luxembourg, Czech Republic, Norway, Germany, and Slovakia. Mink fur farms were planned to be shut-down between 2023 and 2027 in Belgium, Denmark, Netherlands, France, and Bosnia and Herzegovina. COVID-19 accelerated the closure of the mink industry in Europe. Denmark, who initially planned the closure of mink farms in 2023, ordered the slaughter of all animals in 2020 following the massive SARS-CoV-2 outbreak in minks. The same happened in Netherlands with an early closure in 2021. This is unlikely, however, to change the trade choices of other mink-producing countries such as China or Russia who, for evident commercial reasons, do not plan to stop mink farming. As reported by the Moscow Times on 24 November 2020 (Moscow Times, 2020), Russia is currently testing a coronavirus vaccine for minks and domestic animals and will start large scale vaccination on minks. This SARS-CoV-2 veterinary vaccine is likely to be of interest for mink farmers from other countries. In the past decade, China has significantly increased its domestic mink production, becoming the world leading producer of mink pelts. In the United States, the mink fur industry continues to be a source of income, although exports of mink pelts as decreased after reaching a record high of $215.5 million in 2012. Moreover, it is not because minks have been eliminated that the risk of the emergence of variants in susceptible species disappears. SARS-CoV-2 circulates between susceptible species and sooner or later the question of domestic animals (ferrets and other animal such as cats, dogs, or rabbits) will arise with the possibility of new variants emerging from these species (Fenollar et al., 2021; Maurin et al., 2021; Van Aart et al., 2021). Cats are highly susceptible to SARS-CoV-2 infection (Bosco-Lauth et al., 2020; Gaudreault et al., 2020; Leroy et al., 2020; Shi et al., 2020). In a recent investigation on anti-SARS-CoV-2 neutralizing antibodies in 919 companion animals from northern Italy, 3.3% of dogs and 5.8% of cats displayed measurable neutralizing antibody titers (Patterson et al., 2020). There is evidence for nasal shedding of SARS-CoV-2 by infected cats (Halfmann et al., 2020). Although there is currently no evidence that cats or dogs can re-infect humans back, this hypothesis cannot be totally ruled out and measures to be taken in this event must be anticipated. The vaccination of domestic pets and farm animals could be strategy for protecting animals and avoiding owner infection with SARS-CoV-2 variants selected in susceptible animals. Until an effective animal vaccine is available as a general good practice, pet owners and farm staff suspected of, or confirmed with SARS-CoV-2 infection should avoid coming into contact with animals. Appropriate and protective biosafety measures should always be applied when people have contact with groups of animals. Sick animals should be isolated until the end of the contamination period.

SARS-CoV-2 spike protein was predicted to form complexes with host recepto protein orthologs from a broad range of mammals. This work confirms earlier observations indicating that ACE2 orthologs from both *M. erminae* (Qiu et al., 2020) and *M. putorius furo* (Luan et al., 2020b; Devaux et al., 2021) could serve as receptors for SARS-CoV-2. Both experimental infections of *M. putorius furo* and accidental infections of *M. lutreola* and *N. vison* with SARS-CoV-2 have been reported (Beer, 2020; Kim et al., 2020; Molenaar et al., 2020; Oreshkova et al., 2020; Oude Munnink et al., 2020; Richard et al., 2020; Schlottau et al., 2020; Shi et al., 2020), demonstrating that their ACE2 receptors are suitable for virus binding and entry. These results contrast with the conclusion from Damas et al. (2020) who considered that ACE2 orthologs from Merm, Mput, and Mnig displayed a low score for SARS-CoV-2 binding. Indeed, members of the *Mustelidae* family appear as species highly susceptible to SARS-CoV-2. They can be infected by SARS-CoV-2 from other *Mustelidae* and humans, and they can infect other *Mustelidae* and humans with their own SARS-CoV-2. Recently, the SARS-CoV-2 Marseille-4 variant was isolated from animals caged in a mink farm in France (Fournier et al., 2021). SARS-CoV-2, like all other RNA viruses, is evolving through the quasi-species mechanism (Xu et al., 2004; Jary et al., 2020; Karamitros et al., 2020). Variant viruses emerge post-infection under positive selective pressure specific to the host, usually allowing the virus to escape host defense mechanisms (Cali et al., 2005; Martínez et al., 2012; Woo and Reifman, 2012; Van Dorp et al., 2020). Van Dorp et al. (2020) reported recurrent mutations in SARS-CoV-2 genomes isolated from minks including non-synonymous mutations in the RBD of the SARS-CoV-2 spike protein that independently emerged several times but are only rarely observed in human lineages, indicating ongoing adaptation of SARS-CoV-2 to a new host. Furthermore, recombination is also frequently occurring. Phylogenetic studies showed that two independent events of introduction of SARS-CoV-2 into farmed mink populations from humans occurred in northern Europe (Netherlands and Denmark). This involved a D clade virus on one hand and on a G clade virus on the other hand. The selection pressure exerted by the mink immune system was most likely the reason for the selection of the Y453F mutation. Indeed, this was demonstrated by *in vitro* passing of a VSV-SARS-CoV-2 wild type spike in the presence of the RBD-binding REGN10933 monoclonal antibodies which led to the selection of the Y453F mutation conferring resistance to this antibody (Baum et al., 2020; Hoffmann et al., 2021). More recently, the Y453F mutation was reported in a patient with long-term COVID-19 (Bazykin et al., 2021). Of interest is the distribution of the mink SARS-CoV-2 spike sequences within Group 4 into two populations, one bearing only the Y453F and D614G mutations, and the second accumulating many other mutations. SARS-CoV-2 variants reported in minks in Denmark are very likely to be of mink signatures. This could reflect an acceleration of the mutations of the SARS-CoV-2 spike in minks, all *N. vison*, due to mass rearing conditions generating a high population density and a high rate of contact between animals. This could possibly amplify the genetic drift with the emergence of new genotypes in a context of hyperimmunization of a mink population highly exposed to the virus. Although the Y453F variants resist neutralization by the REGN10933 monoclonal antibodies, our investigation of possible antigenic linear peptides in the spike suggests that the Y453F mutation should not affect the binding of most anti-SARS-CoV-2 neutralizing antibodies.

In order to achieve a data-driven approach predicting the antigenicity of candidate vaccines, many teams have sought to model the epitopes of the SARS-CoV-2 spike protein. Predictions are in fact highly dependent on the algorithm and the size of the target peptide, leading often to different results from one study to another. A previous study investigated SARS-CoV-2 spike B-cell epitopes using the ViPR program and reported 23 linear B cell epitopes, including 20 mapping to S2. The three S1-mapping epitopes were, ^310^KGIYQTSN^317^, ^563^QQFGRD^568^, and ^667^GAGICASY^674^. The authors also investigated the existence of discontinuous B cell epitopes but none seemed to involve the amino acid at position 453 (Ahmed et al., 2020). In their study Wang et al. (2020) reported 17 potential linear B-cell epitopes in the spike using the BepiPred 2.0 and the VaxiJen v2.0 algorithms. Two of these epitopes, ^405^DEVRQIAPGQTGKI^418^ and ^441^LDSKVGGN^448^, were located on the RBD. These authors also indicated that the amino acids L452 and R454, which frame the Y453 residue, were involved in discontinuous B-cell epitope recognition. The study by Crooke et al. (2020) who used the DiscoTop algorithm confirmed the prediction of epitopes 405–418 and 441–450 with the finding of a linear epitope ^405^DEVRQIAPGQTGKIADYNYKLPDD^428^ and of the epitope ^440^NLDSKVGGNYN^450^. Another computational study aiming at predicting immunogenic peptides in the SARS-CoV-2 spike protein was reported by Vashi et al. (2020). These authors also used different epitope prediction programs (ElliPro/IEDB and ABCpred servers) than the one used in this study. Their study did not predict the ^355^RISNCVADYSVL^366^ peptide we describe in our work but they did identify 24 other B-cell epitopes. Out of these 24, 6 mapped in the RBD (^314^QTSNFRVQPTES^325^, ^407^VRQLAPGQTGKIADYNYKLPDD^428^, ^437^NSNNLDSKVGG NYN^450^, ^461^LKPFERDISTEIYQAGSTPCNGVEG^485^, ^493^QSYGF QPTNGVGYQ^506^, and ^521^PATVCGPKKSTNL^533^), including 2 framing amino acid Y453. Our study partly corroborates another investigation which analyzed the impact of glycan on B-cell linear epitopes and described six epitopes mapping in the RBD: ^331^NITNL^335^, ^339^GEVFNATRF^347^, ^439^NNLDSKVGGNYN^450^, ^472^IYQAGSTPCNGVEGFNCY^489^, ^498^QPT^500^, and ^527^PKKSTNL VKNK^537^ (Wintjens et al., 2020). None of the T-cell epitopes seem to involve the amino acid at position 453 (Ahmed et al., 2020; Wang et al., 2020). However, a peptide ^444^KVGGNYNYLYRLFR^457^ was described as a T-cell epitope able to be presented in the context of HLA-DPA1 (Vashi et al., 2020). Taken together these studies indicate that the antigenic pattern of the SARS-CoV-2 spike is complex and that further analyses are therefore required to estimate whether viruses bearing the Y453F mutation might be less impacted by human immune defenses. In contradiction with previous assumptions (Starr et al., 2020), Greaney et al. (2021), reported that the mutation Y453F does not strongly affect serum antibody binding.

The evolution of SARS-CoV-2 with increasing changes in the functional domains of the S protein is usually considered capable of affecting diagnostic tests and COVID-19 treatment (e.g., with hyperimmune plasma from COVID-19 convalescent patients). It could also have an impact on the effectiveness of vaccine candidates with a need for update to appropriately stimulate the host immune system against the variant viruses. These questions were also raised about SARS-CoV-2 variants circulating in *Mustelidae*. Our data show that the mink SARS-CoV-2 variants re-infecting humans are not likely to create major concerns about diagnosis or vaccination. The only mink-selected mutation affecting the RBD in the SARS-CoV-2 S protein (Y453F) is expected to have implications for viral fitness (ability to infect humans and animals) and transmissibility (by increasing SARS-CoV-2 affinity for the human ACE2), only. This question must, however, continue to be explored because, recently, Hoffmann et al. (2021) reported that the Y453F mutation in the SARS-CoV-2 spike protein diminished the *in vitro* viral entry inhibition by 7/14 human sera/plasma from convalescent COVID-19 patients. It suggests that at least in a fraction of patients who have recovered from COVID-19, the anti-SARS-CoV-2 spike protein immune response is too weak (or incomplete) to provide full protection against mink-derived SARS-CoV-2 variants. However, it should be emphasized that in these *in vitro* experiments, most serum/plasma from convalescent COVID-19 patients completely neutralized the Y453F mutants at the lowest dilution tested.

## Author Contributions

All authors contributed toward conceiving the manuscript. JD performed the full genomic analysis. RF performed the spike sequences analysis. LP performed the 3D analysis. CD performed the multiple sequence alignment and peptide analysis. CD and RF wrote the manuscript. All authors reviewed and approved the final version of the manuscript.

## Conflict of Interest

The authors declare that the research was conducted in the absence of any commercial or financial relationships that could be construed as a potential conflict of interest.

## Publisher’s Note

All claims expressed in this article are solely those of the authors and do not necessarily represent those of their affiliated organizations, or those of the publisher, the editors and the reviewers. Any product that may be evaluated in this article, or claim that may be made by its manufacturer, is not guaranteed or endorsed by the publisher.

## Footnotes

1
https://www.ebi.ac.uk/Tools/msa/clustalo/

2
https://sourceforge.net/projects/pymol/files/pymol/1.8/

3
https://pymolwiki.org/index.php/APBS

4
bioserv.rpbs.univ-paris-diderot.fr

5
https://web.expasy.org/protscale/

6
http://sysbio.unl.edu/SVMTriP/prediction.php
