# Supplementary figures and images for: Spread of Mink SARS-CoV-2 Variants in Humans: A Model of Sarbecovirus Interspecies Evolution
Source: Front Microbiol. 2021 Sep 20;12:675528. doi: 10.3389/fmicb.2021.675528 (PMC8488371; doi:10.3389/fmicb.2021.675528)

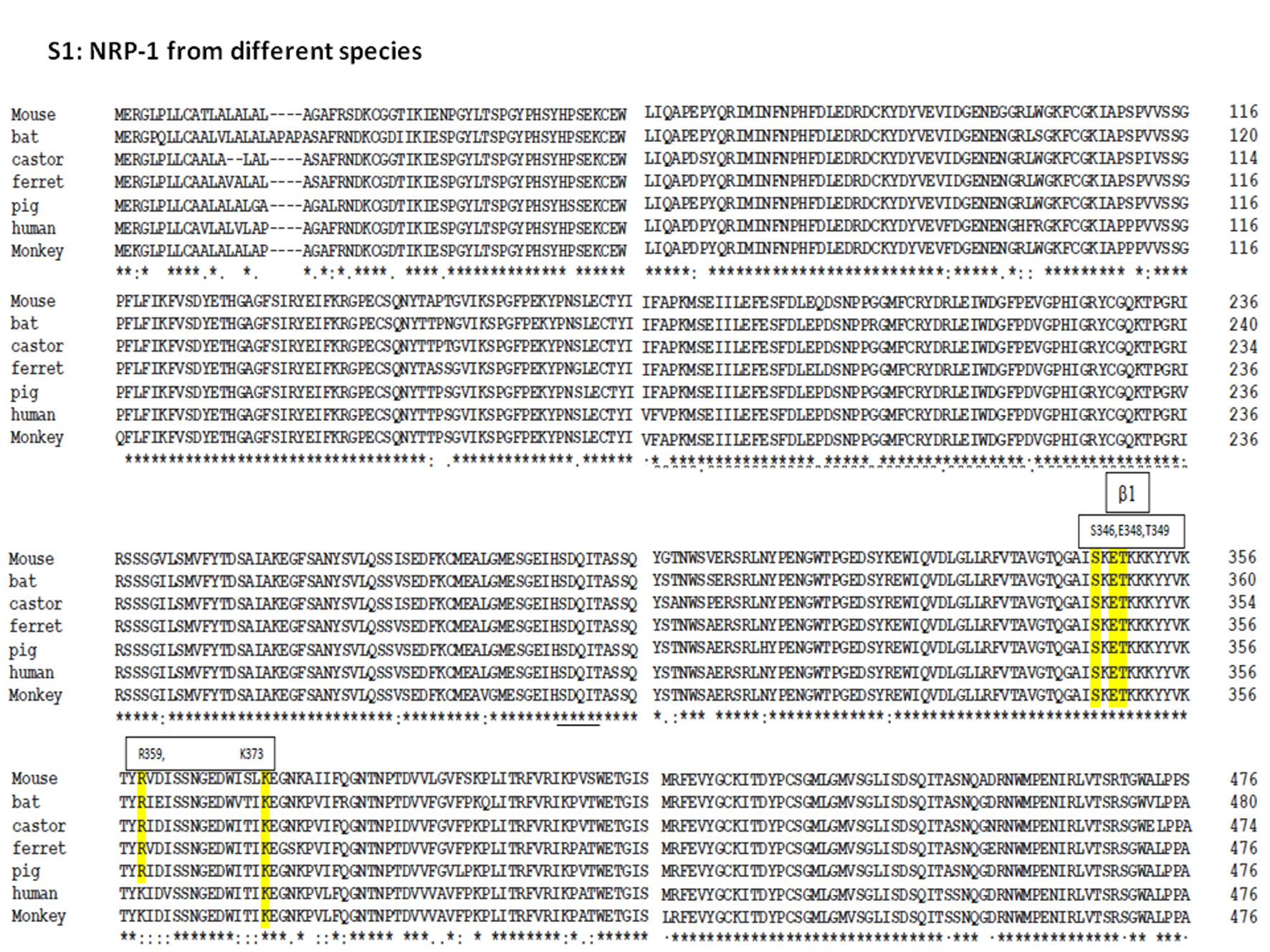

Supplement: Supplementary file 1 [file Image_1.TIF]

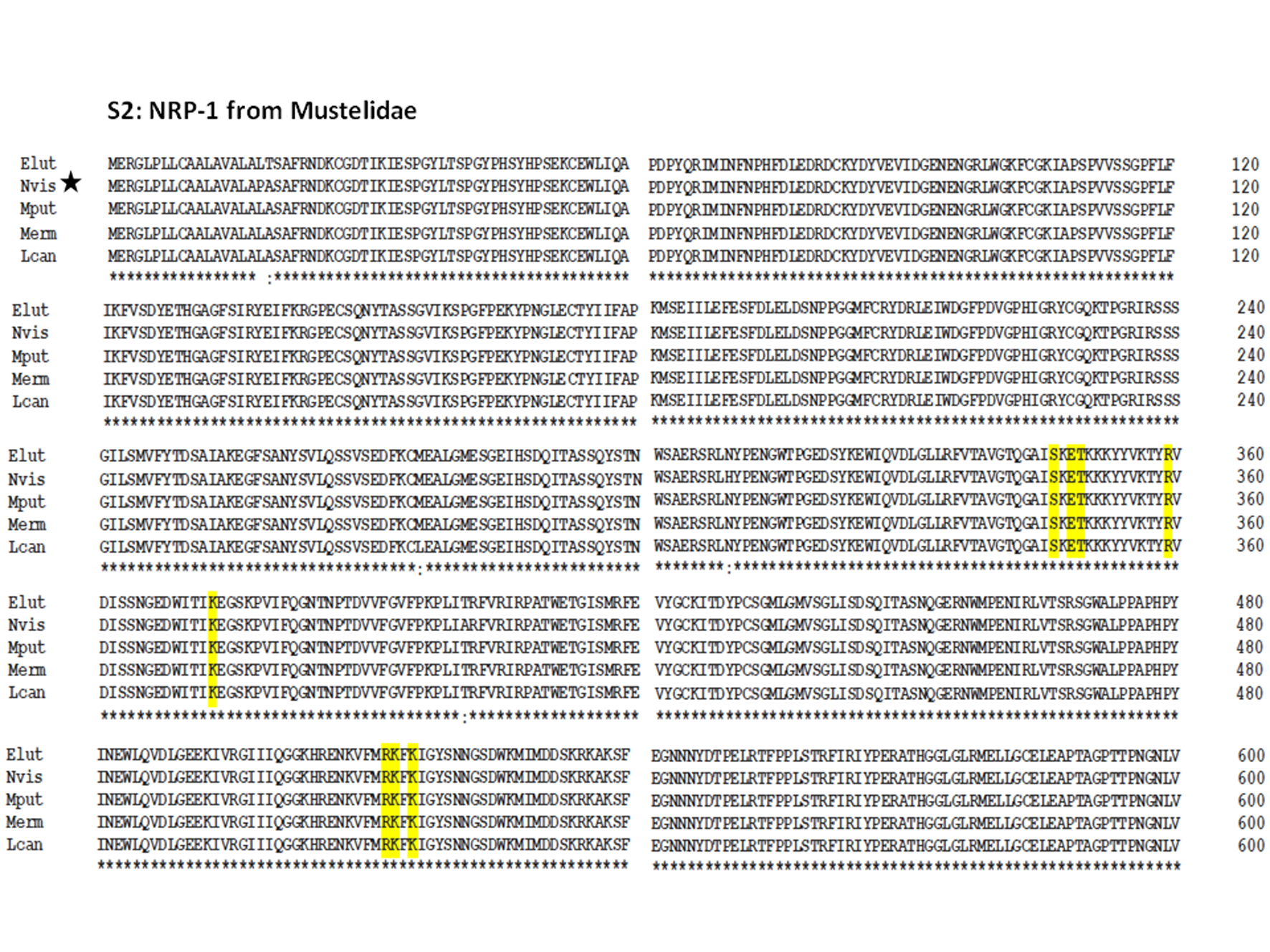

Supplement: Supplementary file 2 [file Image_2.TIF]
